# SynSys: A Synthetic Data Generation System for Healthcare Applications

**DOI:** 10.3390/s19051181

**Published:** 2019-03-08

**Authors:** Jessamyn Dahmen, Diane Cook

**Affiliations:** School of Electrical Engineering and Computer Science, Washington State University, Pullman, WA 99164, USA; jb3dahmen@wsu.edu

**Keywords:** Synthetic data, hidden Markov models, regression, smart homes, healthcare data, activity recognition

## Abstract

Creation of realistic synthetic behavior-based sensor data is an important aspect of testing machine learning techniques for healthcare applications. Many of the existing approaches for generating synthetic data are often limited in terms of complexity and realism. We introduce SynSys, a machine learning-based synthetic data generation method, to improve upon these limitations. We use this method to generate synthetic time series data that is composed of nested sequences using hidden Markov models and regression models which are initially trained on real datasets. We test our synthetic data generation technique on a real annotated smart home dataset. We use time series distance measures as a baseline to determine how realistic the generated data is compared to real data and demonstrate that SynSys produces more realistic data in terms of distance compared to random data generation, data from another home, and data from another time period. Finally, we apply our synthetic data generation technique to the problem of generating data when only a small amount of ground truth data is available. Using semi-supervised learning we demonstrate that SynSys is able to improve activity recognition accuracy compared to using the small amount of real data alone.

## 1. Introduction

When creating models from sensor data, machine learning algorithms need to be trained and validated using diverse datasets, including some with known patterns and distributions. However, many types of real-world sensor-driven datasets are limited in terms of availability and variety. This can introduce difficulties when employing machine learning techniques that rely on large labeled training datasets. In order to address this problem, synthetic data can be created for initial testing and validation of novel machine learning techniques.

In this paper, we introduce a new method for generating synthetic sensor data that is reflective of human behavior found in real sensor datasets. We base the fundamentals of our work on earlier efforts that use machine learning and modeling-based methods to improve the realism of synthetic human behavior data.

**Figure 1:**
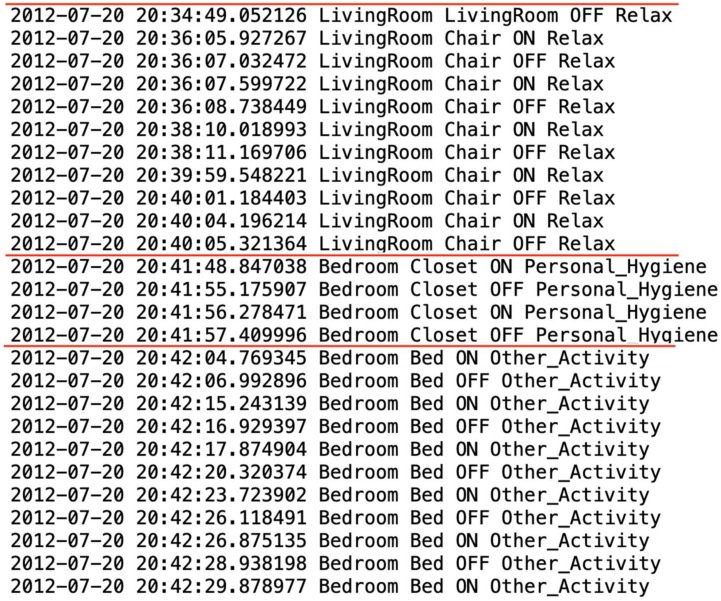
Activity-labeled smart home sensor data that exhibit a nested sequence structure.

We create a novel approach to the problem of generating realistic synthetic smart home sensor data. Our approach employs hidden Markov models (HMMs). Hidden Markov models lend themselves well to the problem of modeling smart home data, due to the data’s sequential nature and a hidden Markov model’s sequence-generative nature. We use real smart home datasets similar to the data shown in Figure 1 to train a model and generate synthetic data.

To validate the realism of data generated using our synthetic data technique, we use data similarity measures to demonstrate that the synthetic data generation technique is not random and that it preserves the underlying patterns and structures of real data while still providing a way to generate arbitrary amounts of new artificial data. We then apply synthetic data generation to the problem of generating synthetic data for semi-supervised activity recognition to improve algorithm accuracy when only a small amount of real annotated data is available. This illustrates how SynSys can be used in a real world machine learning application.

The main contribution of this work is to be able to provide synthetic data that are consistent with the complexities found in real smart home data. This synthetic data can then be used to help test and improve machine learning-based techniques by augmenting small labeled training datasets.

## 2. Related Work

Human activity and behavior data is time consuming and costly to collect and is often limited in terms of availability. Some early efforts to create simulations of sensor-based human behavior data relied on mathematical models such as Markov chains and Petri networks to model daily activities and physiological parameters [1]. Later efforts combined these methods with other modeling approaches to simulate more complex data. Helal et al. used Markov chains to model patterns of activities combined with a Poisson distribution to produce the generated timestamps [2]. This work later evolved into a fully-realized system to help create synthetic datasets and share them with the research community [3]. Other recent work used generative adversarial networks (GANs) to generate data. This approach employs a discriminative model, or adversary, to determine whether generated samples appear to be from the real data distribution. The generative model keeps trying to improve the realism of the generated samples until the adversary cannot distinguish real from synthetic data [4].

When the availability of real ground truth labeled data is constrained, synthetic data can be created to augment the available labeled data in order to more effectively apply machine learning algorithms and techniques that require larger sets of labeled data for effective training. For example, Adar et al. successfully increased classification performance when using GANs to enlarge training data size and diversity for liver lesion classification [5]. Forestier et al. used a weighted version of the time series averaging method, Dynamic Time Warping Barycenter Averaging, to enlarge training time series data sets to increase the accuracy of a 1-NN dynamic time warping classifier [6].

Other methods that have been designed for synthetic data generation run into challenges when creating data that are not i.i.d., and exhibit both high dimensionality and a high level of complexity. For this reason we introduce SynSys to addresses these issues specifically for human-driven sensor time series data. In our experiments we use semi-supervised learning combined with synthetic data generation to demonstrate how SynSys-generated synthetic data can be used to improve the accuracy of machine learning models when the availability of real labeled training data is limited. Semi-supervised learning has often been used to improve the accuracy of machine learning models by making use of available unlabeled data. In the case of medical image analysis, where labeled data is often obtained using human annotators, semi-supervised learning has proven to be able to make use of large amounts unlabeled data to supplement the small amount of labeled data to improve classification accuracy [7]. Semi-supervised learning has also been used for the problem of disease diagnosis from large amounts of often unlabeled biomedical data [8].

## 3. Smart Home-Based Activity Data

The goal of this work is to create synthetic data that is consistent with real-world, activity-labeled smart home sensor data. Activity-labeled smart home sensor data has unique characteristics in that it is multivariate, contains spatio-temporal relationships, and is reflective of dynamic human nature. Furthermore, due to the fact that each data point contains time and activity information in addition to a sensor reading, this is time series data that exhibit a hierarchical organization. These data contain a sequence of data points, each of which is comprised of its own sequence. In our case, activity-labeled smart home sensor data can be represented as time series data or a sequence of time-stamped sensor readings (or sensor event data). Figure 1 shows a snippet of smart home data. As Figure 1 shows, smart home data can be represented as a structured time series containing a series of sensor events, ordered by time, that represent activities occurring in a smart home. If we divide the sensor events into blocks of activities, or segment the data as indicated by the red lines, we can also observe a higher level sequence within the data, namely the sequence of activities. In this paper, we focus on generating synthetic data that emulates this type of nested sequence structure. This type of data is multivariate, non-i.i.d., and contains sufficient complexity and spatio-temporal dependencies to warrant a new approach for synthetic data generation.

The real smart home data we use in our experiments are collected from older adult participants with CASAS smart home in a box (ShiB) sensors installed in their homes [9]. Table 1 provides summary statistics for the smart home sites used in our experiments. In CASAS smart homes, as the resident moves around the home performing daily activities, sensor events are recorded that indicate the time of sensor activation as well as other information about the originating sensor. This information is then used in AR [10], an activity recognition algorithm which is trained from human-annotated ground truth activity data truth to label sensor events with a label from the following set of activities: *Cook*, *Eat*, *Sleep*, *Personal Hygiene*, *Take Medicine*, *Work*, *Leave Home*, *Enter Home*, *Bathe*, *Relax*, *Bed Toilet Transition*, *Wash Dishes*, and *Other Activity*. The types of sensors that we use for our experiments include passive infrared motion sensors which activate when heat-based movement occurs within their field of view and door sensors which are magnet pairs that activate when a door is open or closed.

AR labels each sensor event with the activity that was performed at the time the event was observed. To generate an activity label for smart home sensor events, AR uses a windowing approach for feature vector creation. Let *S* = *s*_1_, *s*_2_, …, *s_N_* represent the sequence of sensor events. The sequence *S* is divided into fixed-size windows. A window *W_j_* can then be represented by the sequence [*s_j_*, *s_j_* + *∆s*] where *∆s* varies depending on experimental context. Once *W_j_* is defined, features $x\in {ℜ}^{d}$ are extracted from the sensor data input. This feature vector *x_j_* captures the time of the first and last sensor events, the temporal span of the window *W_i_* and a count of the different sensor events within the window. A summary of features that are extracted for activity recognition and for activity prediction is provided in Table 2.

## 4. SynSys Design

In this section we provide an overview of the components that make up SynSys. SynSys is available for download online and is supported by the pomegranate Python library for HMMs (SynSys: https://github.com/jb3dahmen/SynSys-Updated, pomegranate: https://github.com/jmschrei/pomegranate).

### 4.1. SynSys System Overview

Smart home data can be represented as a structured time series containing a series of sensor events, ordered by time, that represent activities occurring in a smart home. Each sensor event contains the event timestamp, the sensor name, the sensor state and the activity label. Upon closer inspection of the timestamps we can see that this time series data does not have equal time intervals between data points. This is due to the fact that each instance of the data represents a sensor event which is triggered by residents moving around the smart home space and activating the corresponding sensor. The sensors used in this case are ambient discrete event sensors. As a result, they generate text-based messages report their state when the state of the sensed environment changes, in contrast with polling sensors that report their state at equal time intervals. In order to generate synthetic data that resembles this kind of time series we divide the problem into three separate learning tasks.

- Generating a realistic sequence of activities,
- Generating a realistic sequence of sensor events within an activity, and
- Generating realistic timestamps that capture the duration of sensor event occurrences.

We describe each of these learning problems in detail and then provide an explanation for the overall system that can be used to produce realistic synthetic data of this type. In order to generate realistic synthetic data we combine the strategies for each of the learning tasks described above into a system called SynSys. We first train an HMM to generate a realistic sequence of activities (the outer sequence).

Next, we train a separate HMM for each activity. These second-level HMMs generate a sequence of sensor events reflective of the activities they represent. Once the first-level HMM generates a sequence of activities, each activity is expanded into a corresponding sequence of sensor events by the corresponding second-level HMM. We refer to these as the nested sequences. Finally, we train regression learners to create realistic timestamps that capture the time gaps between sensor events and the duration of each activity.

**Figure 2:**
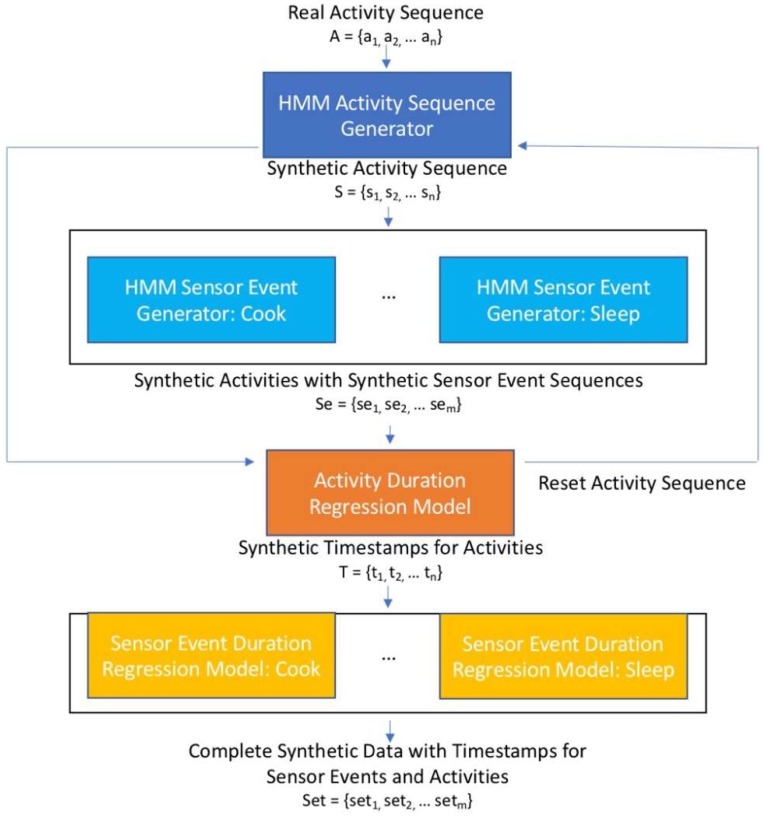
Overview of the SynSys system for example activities Cook and Sleep.

Figure 2 shows an overview of SynSys. While we applied HMMs to the problem of generating sequences at each step, this system can be generalized to employ any sequence generation technique other than HMMs at the sequence generation steps 1 and 2 shown in Figure 2. Additionally, while we use SynSys specifically to generate synthetic smart home data, the method can be used to generate hierarchical time series data for a number of real-world applications. Such applications include sentiment analysis, music genre analysis, analysis of web traffic, and analysis of public transportation systems [11,12,13].

### 4.2. Generating a Realistic Activity Sequence 

In Figure 1 we can see blocks of sensor events that have the same activity label. These blocks represent a series of sensor events that comprise a single smart home activity occurrence. In order to capture only the sequence of activities that occur in a smart home we can remove the sensor event information and replace each activity block with a single data point, labeled by the corresponding activity. The start of the activity is defined as the first sensor event with activity label *A* and the end of the activity is the last sensor event with activity label *A*. If a different activity label is encountered a new activity is recorded. Using this simplified version of the data we can then use probabilistic modeling techniques to learn the sequence patterns of activities from real data and then generate new synthetic sequences of activities based on the learned model. To generate these sequences we use a 12 state hidden Markov model. This size was selected based on empirical evidence from our smart home data.

### 4.3. Handling Other Activity and Enforcing Day Structure

In our real smart home data sets, an activity label called “Other Activity” is used to encompass all unknown activities. When manually annotating data with ground truth activity labels, a set of known activities are detected and labeled. These activities correspond to categories often used in assessment of basic or instrumental activities of daily living (ADLs) [14]. However, these predefined activity categories typically make up approximately half of the total sensor data. These findings are consistent with survey-based instruments revealing that less than half of reported time use falls into these well-understood activity classes [15]. This results in a large number of Other Activity labels being present in the real data. The prevalence of Other Activity occurrences in smart home data will bias the HMM toward inserting Other Activity labels into the activity sequence not only often, but in unintuitive sequences.

We hypothesize that the Other Activity generation error is due to the fact that this activity actually represents a large collection of actual, although unlabeled, smart home activities. To address the resulting bias issue, we add a preprocessing step to SynSys that decomposes the Other Activity class into clusters representing smaller activities. To achieve this we used a K-means clustering approach that used Euclidean distance with K = 12 clusters on the sensor event sequence data with Other Activity to remap the Other Activity label to a new label from the set {C1, C2, …, C12}. The hyperparameter K = 12 was selected to generate activity cluster sizes that approximate the mean frequency of the predefined activity classes. Table 3 shows a summary of the frequency means and standard deviations for the predefined activity classes of sites used in our semi-supervised activity recognition experiments. Once the synthetic data is generated, the cluster labels can be replaced with Other Activity labels for the final generated synthetic data to best resemble real smart home data.

A basic HMM represents a sequence of symbols, which in this case are activity labels. After being trained on a simple sequence of actual smart home activities, the HMM structure does not reflect the gaps in time that occur between one activity and the next. The resulting generated sequence of synthetic activities produced some activities that were overly long and others that were unusually short. The activity timing error propagated downstream and over the course of a day or week looked less like the real sequence data. To address this and enforce a more realistic day like structure, at the timestamp generation step for the sequence of activities we included a reset step that occurs after every *x* time units. The reset period, *x*, can be determined by the user. For our synthetic data generation we set *x* to one hour.

To periodically reset the data sequence, SynSys identifies the high-probability activities to occur at the beginning of each reset period. When the beginning of the reset period is reached, SynSys compares the synthetically-generated activity label with the highest-likelihood activity label. If there is a mismatch, SynSys resets the start state of the HMM data generator to be the highest-probability activity state and continues with the data generation process from that state replacing segments of data generated using the old activity label. By performing this reality check as we generate timestamps, we are able to help the HMM activity generation produce a more realistic sequence of activities that occur each day.

### 4.4. Generating a Realistic Sequence of Sensor Events Within an Activity

Within each activity block shown in Figure 1 is a sequence of actual sensor events. In order to generate realistic sequences of sensor events based on activities we train a separate generative model for each activity type. Again, SynSys utilizes hidden Markov models by training a separate model for each activity with corresponding sensor event sequences from real data. Using the previously-generated sequences of activities, SynSys then expands an activity instance into its corresponding sequence of sensor events. To do this, SynSys chooses a random number from the distribution of actual sensor event lengths for the corresponding activity as the number of sensor events to generate for this activity occurrence.

### 4.5. Generating Realistic Timestamps

Once we have generated the activity sequences and sensor events, all that is left to complete the synthetic data is to generate the timestamp information. These timestamps in the collective represent the durations of each activity and in the individual represent the time gaps between each sensor event that occurs within an activity. In order to learn realistic timestamps, SynSys employs a ridge regression machine learning approach to better learn how to capture a time-based representation of events. Ridge regression is a technique for analyzing multivariate regression data that exhibits near-linear relationships among the independent variables [16]. Using ridge regression the loss function is represented by the linear least squares function and regularization is given by the L2 norm. This helps limit the complexity of the regression model and multi-collinearity which is a preventative for over-fitting. First, a ridge regression model is trained from actual smart home data to learn the durations of each activity. Next, separate regression models are trained from smart home data to learn the time gaps between sensor events within each activity. While HMMs are well suited to learning sequence orders, applying HMMs to the problem of learning non-uniform durations adds complexity to the model that may not produce intuitive results. We hypothesize that regression models will be effective at learning more complex timing information.

In order to train a model that learns the durations of each activity we construct a feature vector from the real data. The vector is *x* = <*activity*, *time of day*, *day of week*, *current hidden state*, *previous hidden state*> and the corresponding target variable *y* represents the duration of each activity in microseconds. The synthetic activity sequence provides the activity and day of week features for *x*. The timestamp for the first synthetic activity is the specified time provided by the real smart home data. Next, this new observation is input to the learned regression model to produce a predicted duration. We then add this duration to the first timestamp to obtain the timestamp for the next activity in the sequence. While generating the activity durations, SynSys checks whether the synthetic activity matches the most probable activity at the current time of day as described previously. If the activity does not match, we generate a new portion of the synthetic activity sequence using the state that is most likely to emit the most probable activity as the start state for synthetic sequence generation. We continue this process until timestamps have been generated for all activities in the sequence and the sequence has been reset every hour to represent a more realistic order of activities for each day in the sequence.

To train each model to learn the time gaps between each sensor event we construct a similar feature vector *x* = <*sensor event*, *time of day*, *day of week*, *current hidden state*, *previous hidden state*> from the real data where the “sensor event” consists of both the sensor name and the corresponding state. We then repeat the process of fitting a regression model for each type of activity to this data to output the time gaps between each sensor event. To obtain synthetic timestamps for each synthetic sensor event for each activity we use the time that was predicted for the synthetic activity start as the first time for the first sensor event and then learn the time gaps for each of the subsequent synthetic sensor events within that activity using the corresponding model. If the time gaps of the sensor events starts to extend beyond the timestamp for the next activity we cut off the current sensor event sequence to give the durations between activities dominance in the data hierarchy.

## 5. Methods

### 5.1. Similarity Measures

In order to examine the realism of our SynSys synthetic data generator we first compare one week of randomly-chosen real data with one week of synthetically-generated data for the same home and same time period using two common measures of distance for time series data, Euclidean distance and Dynamic Time Warping (DTW). We do this for each of the 10 smart home datasets summarized in Table 1 and report the average distance results. Euclidean distance, DTW, and their variations are established distance measures that have been used compute the distance or similarity between two time series datasets in a wide variety of research studies and applications [17,18,19]. To generate the synthetic data we use SynSys HMMs with 12 states for activity and sensor event sequence generation, a one hour reset period, and a ridge regression algorithm for timestamp generation. As a baseline we create a random version of the data by randomly permuting each sensor event within the real data. The original timestamps are kept in their original order and each piece of the content of the sensor event is randomly permuted, i.e., each line of random data will contain the original timestamp and then a randomly-selected <*sensor event name*, *state*, *activity label*> tuple from the original data. The distance values reported in our results are measures of dissimilarity meaning that larger values indicate more differences between datasets.

To further examine the realism of the data we also compute the distances between one week of real data and one week of real data from another home with the same time period as well as the distances between one week of real data and week data of real data from the same home during another time of year. We assume that the activity patterns of the smart home resident changes at different times of year (for example the resident may be more likely to stay inside during winter vs. summer or have a different schedule year to year) and that the activity patterns of residents will differ between homes.

### 5.2. Real World Application

To demonstrate how synthetically-generated data can be used to improve the performance of machine learning algorithms when real labeled data is scarce we use SynSys combined with semi-supervised activity recognition to augment a real smart home dataset. We hypothesize that training an activity recognition algorithm with the synthetically-augmented data will boost performance of the recognition algorithm.

To perform semi-supervised learning, we first train AR on subsets of data extracted from one month of ground truth-annotated sensor-based activity data for 10 smart home sites. To modify AR to be semi-supervised we use a self-training approach. In this approach the classifier is first trained with a small amount of labeled data and is then used to provide labels for the unlabeled data. The original unlabeled data is combined with the predicted labels and added to the training set. The classifier is retrained using this newly-labeled training set and then tested only on the original data with ground truth labels. AR is trained to recognize classes from the set of activities described in Section 1. To assess the impact of adding synthetic data, we vary the amount of real data that is fed to AR in terms of the number of sensor events that are included in the training data. The amount of synthetic data is constant in each case and corresponds to one month of generated data. The final 200,000 sensor events, approximately three weeks of real data, are held out for testing. We report the accuracy averaged over the 10 smart home sites.

## 6. Results

Table 4 summarizes the average distances between one week of real data and the randomly-generated data as well as the average distances between one week of real data and the synthetically-generated data using the 10 smart home datasets summarized in Table 1. To provide a baseline that reflects the natural variability between different smart homes, Table 4 also shows the average distances between one week of real data and one week of real data from another home as well as the average distances between one week of real data and week data of real data from the same home during another time of year. Furthermore Table 4 shows the distance between one week of real data and one week of synthetic data based on a simplified single HMM and Poisson regression. This is intended to demonstrate how SynSys would compare to an alternative synthetic data generation method that does not use combinations of HMM’s, Ridge Regression, and a reset period to enforce day structure. A higher number indicates that there is a greater distance between the data, meaning the data is more dissimilar. For generating a week of synthetic data the average runtime across the 10 datasets was 49.18 s.

Table 4 summarizes the average accuracy for semi-supervised activity recognition for the 10 smart home datasets summarized in Table 1 for different sized subsets of training data in terms of number of sensor events or lines. Table 5 shows the accuracy first using only the real data and then the accuracy using the real data enlarged with a month of synthetically-generated data.

## 7. Discussion

The results from Table 4 provide evidence that the SynSys algorithm was able to successfully generate more realistic synthetic data for a week of smart home data in comparison to random data, data from another home, and data from another time period. Our data exhibited a dominant activity, Other Activity, that encompassed a wide variety of sensor events and was very prevalent in our data. We found that including a preprocessing step to break apart this activity into smaller clusters helped the performance of the HMM based sequence generation by not biasing it towards including Other Activity. This may indicate that other datasets that are very unbalanced in terms of the sequences that need to be generated with a portion of the sequence that is “Other” or “Unknown” may also need to include some preprocessing steps in order for the SynSys approach to be more effective.

Such realistic data can be used to boost the performance of machine learning algorithms through semi-supervised learning. In the case of AR, the results from Table 5 demonstrate that SynSys synthetic data can be used to augment limited labeled smart home data. This method of self-training does improve the performance of a semi-supervised activity recognition algorithm.

Because SynSys is based on generating nested sequences of data and sequences of timestamps that capture duration, the method can be applied to more generalized data beyond the smart home datasets we used in our experiments. One example of this could be to apply this approach to natural language datasets that exhibit this kind of nested sequence structure with different parts of a language’s grammar. The synthetic data can then be used to train and test the performance of novel machine learning algorithms that require larger labeled data sets that are expensive and time consuming to obtain.

## 8. Conclusions

We discussed a novel approach to synthetic data generation using a combination of HMMs and regression algorithms. We demonstrated that our approach was able to generate more realistic data in comparison to random data, data from another home, and data from another time period. We also provided a real world application of our approach using and experiment examining the accuracy of semi-supervised activity recognition with a synthetically augmented smart home dataset. We discussed how this approach could be generalized to generate data for a wider range of data sets. In the future we hope to use SynSys a method of generating large amounts of normal and outlier detection for the purpose of testing anomaly detection techniques for health event detection.

## Figures and Tables

**Table 1:** Summary statistics for each of the smart home samples used in our experiments.

Dataset	Number of Residents	Number of Days	Total Number of Motion Sensors	Total Number of Door Sensors
1	1	6	16	1
2	1	5	23	3
3	1	10	10	1
4	1	4	25	4
5	1	6	18	4
6	1	6	24	4
7	2	3	24	1
8	1	4	22	3
9	1	3	17	4
10	1	5	15	4

**Table 2:** Summary of activity recognition features.

Feature	Description
lastSensorEventHours	Hour of day
lastSensorEventSeconds	Seconds since midnight
windowDuration	Length of window (seconds)
timeSinceLastSensorEvent	Seconds since previous event
prevDominantSensor1	Most frequent sensor in previous window
prevDominantSensor2	Most frequent sensor two windows ago
lastSensorID	Most recent sensor identifier
lastLocation	Most recent sensor location
sensorCount	Number of events in window for each sensor
sensorElTime	Elapsed time since each sensor fired

**Table 3:** Summary statistics of activity occurrence frequencies for the smart home data sets used in our experiments. Datasets contained an average of 227,511 sensor events with a standard deviation of 93,572.

Predefined Activity Label	Mean Frequency	Standard Deviation
Sleep	10,075	9400
Other Activity	96,762	53,116
Bed Toilet Transition	1949	2497
Personal Hygiene	20,646	14,229
Take Medicine	2463	1870
Relax	17,878	15,032
Eat	11,873	7654
Work	12,208	12,325
Leave Home	1708	867
Enter Home	1561	629
Wash Dishes	13,908	7375
Cook	33,915	15,946
Bathe	2561	3275

**Table 4:** Distances between real data, synthetic data, and randomly generated data. Clustering is employed to group events within the Other Activity category. * = difference between SynSys and Real and other approach is statistically significant (*p* < 0.05).

Data Comparison	Euclidean	DTW
Random and Real	2048 *	364,392 *
Real and Real from Another Home	1799 *	302,221 *
Real and Real from Another Time Period	1781 *	311,438 *
Real and Simple HMM&Poisson	1846 *	310,964 *
SynSys and Real	1474	194,011

**Table 5:** Activity recognition accuracies, using semi-supervised learning with a varying amount of real data and one month of synthetic data. * = difference from accuracy trained on real data is statistically significant (*p* < 0.05). The list of activities learned is mentioned in the introduction.

Number of Real Sensor Events used for Training	Accuracy When Trained on Real Data Only	Accuracy When Trained on Real Data Plus 200,000 Synthetically-Generated Sensor Events
5000	35.54% ± 5.10%	47.90% * ± 3.60%
10,000	45.50% ± 5.75%	61.19% * ± 4.96%
15,000	41.68% ± 5.49%	52.83% * ± 3.23%
20,000	47.61% ± 5.91%	57.57% ± 2.99%
